# A Pan‐Cancer Atlas of TIPE2 Identifies Its Association With the Tumor Immune Microenvironment, Prognosis, and Immunotherapeutic Potential

**DOI:** 10.1155/humu/7558801

**Published:** 2026-04-15

**Authors:** Hui Cao, Wenyu Jia, Hao Yang, Yuling Zhang, Shougen Cao, Yanbing Zhou, Zequn Li

**Affiliations:** ^1^ Department of Gastrointestinal Surgery, The Affiliated Hospital of Qingdao University, Qingdao, Shandong, China, qdu.edu.cn; ^2^ Department of Endocrinology, Qingdao Municipal Hospital, Qingdao, Shandong, China, qdslyy.cn; ^3^ Department of Colorectal and Anal Surgery, Weifang People′s Hospital, Weifang, Shandong, China, wfph.cn; ^4^ Gastrointestinal Tumor Translational Medicine Research Institute of Qingdao University, Qingdao, Shandong, China

**Keywords:** immune cell infiltration, immune checkpoint gene expression, pan-cancer analysis, prognostic biomarker, TIPE2 (TNFAIP8L2), tumor microenvironment

## Abstract

The tumor microenvironment (TME) plays a pivotal role in tumorigenesis, progression, and metastasis. Tumor necrosis factor alpha–induced protein 8‐like 2 (TNFAIP8L2; TIPE2), a member of the TIPE family, is a key regulator of immune homeostasis. In this study, we comprehensively characterized the pan‐cancer landscape of TIPE2 and evaluated its potential significance for prognosis and immunotherapy‐related stratification. Using multiomics data from public databases such as TCGA, GTEx, and HPA, we systematically analyzed TIPE2 expression patterns, genetic and epigenetic alterations, and their associations with clinical outcomes and immune characteristics across 33 cancer types. TIPE2 was found to be enriched in immune cells and frequently dysregulated in tumors compared with normal tissues. TIPE2 expression demonstrated a significant correlation with the infiltration of various immune cell populations across most cancers and exhibited strong associations with the expression of immune checkpoint genes. Alterations in TIPE2, along with aberrant methylation, were identified in a subset of tumors, indicating multilayered regulatory mechanisms. Gene set enrichment analysis further demonstrated that elevated TIPE2 expression is associated with immune‐related pathways. Survival analyses demonstrated that TIPE2 expression was associated with overall survival and disease‐free survival across multiple cancer types, exhibiting directionally heterogeneous effects depending on tumor context. Collectively, these findings identify TIPE2 as an immune‐relevant biomarker candidate with prognostic significance across various cancers and warrant further mechanistic and clinical validation to elucidate its potential role in immunotherapy‐based patient stratification.

## 1. Introduction

Each generation of malignant tumor cells is frequently accompanied by gene mutations, whereas tumor enlargement and metastasis occur in conjunction with changes in the tumor microenvironment (TME). The TME, which plays a significant role in tumorigenesis and progression, constitutes a complex ecological system comprising malignant cells and reprogrammed surrounding cells influenced by various cytokines, chemokines, and other factors secreted by the malignant cells [1]. Among these, immune cells play a crucial role in tumor progression owing to their involvement in the inflammatory response. Cancer progression is believed to result from the combined influence of tumor cells, immune infiltrating cells, fibroblasts, and other surrounding cells [2–4].

Tumor necrosis factor alpha‐inducible protein 8‐like 2 (TNFAIP8L2, TIPE2) is a member of the tumor necrosis factor alpha‐inducible protein 8 (TNFAIP8) family. Previous studies have demonstrated that TIPE2 serves as a crucial negative regulator of Toll‐like receptor (TLR) and T‐cell receptor (TCR) functions and is essential for maintaining immune homeostasis [5–11]. Furthermore, due to the high homology in the spatial structures of TIPE family members, TIPE2 also functions as a phosphoinositide transfer protein that guides leukocyte polarization and directional migration [12–15]. In addition, TIPE2 plays a distinctive role in the initiation and progression of various types of tumors [16–20].

In this study, we utilized multiomics public data encompassing transcriptomic, genomic, epigenetic, and clinical dimensions to construct a systematic and comprehensive Pan‐Cancer Atlas of TIPE2. The uniqueness of this study resides in its multilayered analytical framework, which correlates TIPE2 expression across 33 tumor types with DNA methylation status and mutation landscapes to elucidate shared regulatory mechanisms across diverse histological origins. Specifically, we employed these integrated datasets to (1) comprehensively evaluate TIPE2 alterations across multiple cancer types, (2) elucidate the relationships between TIPE2 alterations and tumor‐infiltrating immune cells as well as associated immune markers, and (3) visualize the resulting prognostic implications for cancer diagnosis and treatment. Our analysis offers a comprehensive understanding of the role of TIPE2 in tumors and contributes to the advancement of existing immunotherapeutic strategies [4, 21].

## 2. Methods

### 2.1. cBioPortal (https://www.cbioportal.org/)

We conducted TIPE2 gene alteration analysis utilizing cBioPortal, an open network platform based on the TCGA database that integrates data mining, data integration, and analysis [22].

### 2.2. Gene Set Enrichment Analysis (GSEA)

GSEA was employed to assess the enrichment of differentially expressed genes across several predefined pathways. RNA‐sequencing expression profiles and associated clinical data for patients across 33 cancer types were obtained from the TCGA dataset. GSEA Version 4.2.3 software was utilized to identify pathways associated with tumor TIPE2 expression levels [23].

### 2.3. GEPIA2 (http://gepia2.cancer‐pku.cn/)

GEPIA2 is a tool for gene expression correlation analysis that extensively utilizes tumor and normal sample data from the TCGA and GTEx databases. We utilized this tool to compare TIPE2 expression differences between tumor and normal tissues and to conduct Kaplan–Meier survival analyses of pan‐cancer patients. Simultaneously, we developed a correlation analysis map based on the pathological stage [24].

### 2.4. Human Protein Atlas (HPA) database (https://www.proteinatlas.org/)

The HPA database is a publicly accessible resource encompassing transcriptomic, proteomic, and systems biology data. We utilized the HPA database to analyze the expression levels of TIPE2 mRNA across various tissues and cell types in tumor patients [25].

### 2.5. Tumor Immune Estimation Resource (TIMER) (https://cistrome.shinyapps.io/timer/)

The TIMER web server is a platform designed for analyzing immune cell infiltration across various tumor types. This system was employed to conduct a pan‐cancer analysis of the correlation between TIPE2 expression and immune cell infiltration [26].

### 2.6. UALCAN (http://ualcan.path.uab.edu/)

The UALCAN database is an online platform for data analysis based on cancer omics data, integrating information from databases such as TCGA, MET500, CPTAC, and CBTTC. We employed UALCAN to compare the differences in TIPE2 expression between tumor and normal tissues at the protein level. Methylation analysis of the TIPE2 gene was likewise conducted using this method [27].

### 2.7. The Relationship Between TIPE2 Expression, Immune Cell Infiltration, and Immune Checkpoints

RNA‐sequencing expression profiles, standardized as FPKM‐UQ values, along with corresponding clinical data for patients with 33 types of cancer, were obtained from the TCGA dataset (Pan‐Cancer Atlas 2018 version) through the UCSC Xena browser (https://xenabrowser.net/). To evaluate the reliability of our immune score assessment results, we employed the immunedeconv R software package [28], which incorporates the TIMER and xCELL algorithms [26, 29]. We extracted the expression levels of eight transcripts (CD274, CTLA4, HAVCR2, LAG3, PDCD1, TIGIT, BTLA, and SIGLEC15) associated with immune checkpoints and analyzed the expression values of genes related to immune checkpoints [30]. Spearman′s rank correlation was used to assess associations between TIPE2 expression and immune infiltration metrics, immune checkpoint gene expression, and genomic markers. All analytical methods and R packages were executed using R Version 4.0.3. Unless otherwise specified, two‐group data were analyzed using the Wilcoxon test. *p* values less than 0.05 were considered statistically significant. Given the exploratory nature of this pan‐cancer study, *p* values were not adjusted for multiple comparisons and should be interpreted cautiously.

## 3. Results

### 3.1. Expression of TIPE2 in Various Tumor and Normal Tissues

To explore TIPE2 expression across various normal and tumor tissues, we utilized the GEPIA2 and HPA databases to conduct a comprehensive analysis of TIPE2 mRNA expression levels. As shown in Figure 1a, TIPE2 was expressed in 14 human organs or tissues, with lymphoid tissues exhibiting significantly higher expression levels compared with other tissues and organs [31]. Moreover, TIPE2 expression levels in 31 tumor tissue samples were found to be comparable with or higher than those in normal tissues [21]. The expression levels of TIPE2 in GBM, KIRC, KIRP, LAML, LGG, OV, PAAD, SKCM, and TGCT were significantly elevated compared with those in normal tissues (Figure 1b) [31]. Conversely, analysis using the UALCAN database reveals increased TIPE2 protein expression levels in certain tumor tissues compared with normal tissues (Figure 1c). To further elucidate the causes of these variations in TIPE2 expression, we conducted an analysis of TIPE2 expression at the cellular level. As shown in Figure 1d, TIPE2 is abundantly expressed in both blood and immune cells, clearly demonstrating its significance within the immune system [32].

Figure 1TIPE2 expression varied across different human organs and tissues. (a) Expression of TIPE2 in various human organs and tissues. (b) Differences in TIPE2 mRNA expression between tumor and normal tissues. (c) Differences in TIPE2 protein expression between tumor and normal tissues. (d) TIPE2 expression in all types of cells studied.

**Figure:**
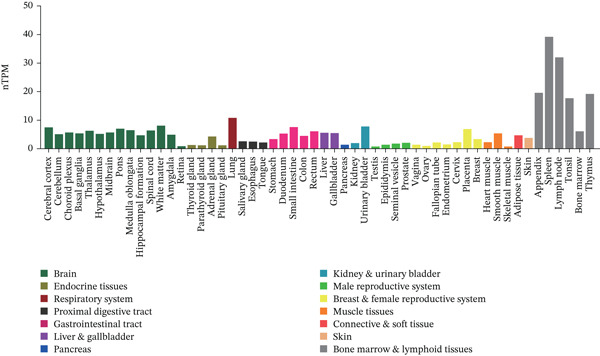
(a)

**Figure:**
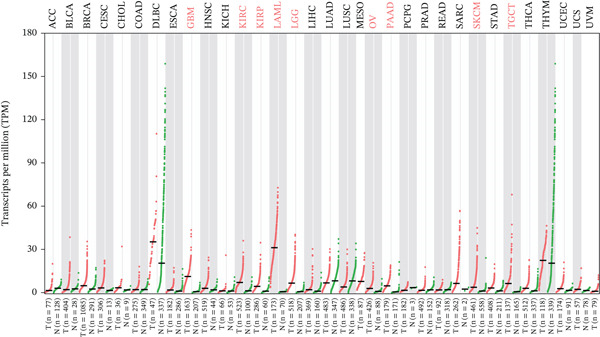
(b)

**Figure:**
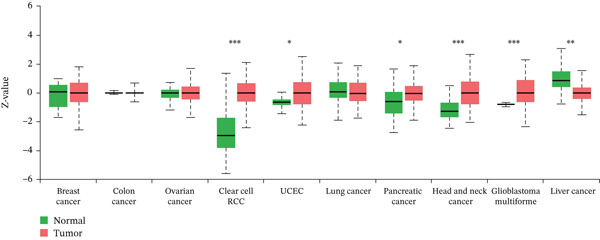
(c)

**Figure:**
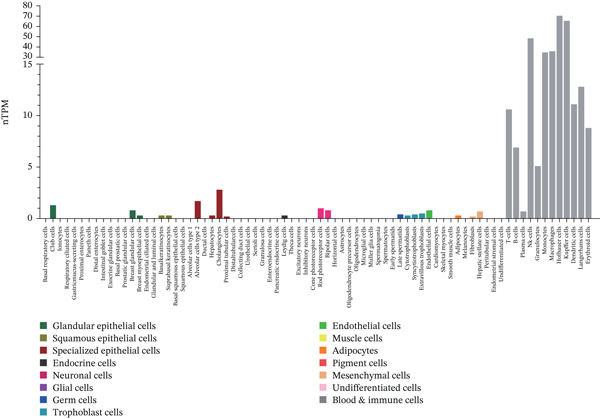
(d)

### 3.2. Correlation Between TIPE2 Expression and Immune Infiltration, Immune Checkpoints, and Genomic Markers

Considering the widespread expression of TIPE2 in immune cells, we investigated the pan‐cancer association between TIPE2 expression and the overall immune landscape [33]. As shown in Figure 2a, correlation analysis revealed that, except for DLBC and UVM, the extent of immune cell infiltration significantly correlated with TIPE2 expression [11, 31, 32]. To further explore this, we employed xCell and found that TIPE2 is strongly and positively correlated with immune infiltration across nearly all tumor types (Figure 2b). Similarly, as shown in Figure 3a, TIPE2 expression exhibited widespread positive correlations with the majority of immune checkpoint genes across most TCGA cohorts, except for DLBC and LAML. Furthermore, given that MSI and TMB are novel markers associated with the immunotherapy response, we investigated their relationship with TIPE2 expression and identified clear correlations solely in individual tumors (Figure 3b,c) [30, 33, 34].

Figure 2Correlation between TIPE2 expression and immune cell infiltration. (a) TIPE2 expression demonstrated a significant correlation with the infiltration levels of various immune cells based on the TIMER database. (b) TIPE2 expression was significantly correlated with the infiltration levels of various immune cells based on xCell.

**Figure:**
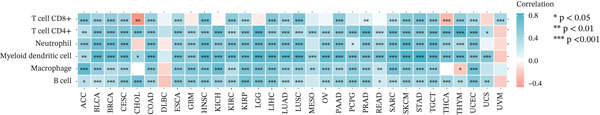
(a)

**Figure:**
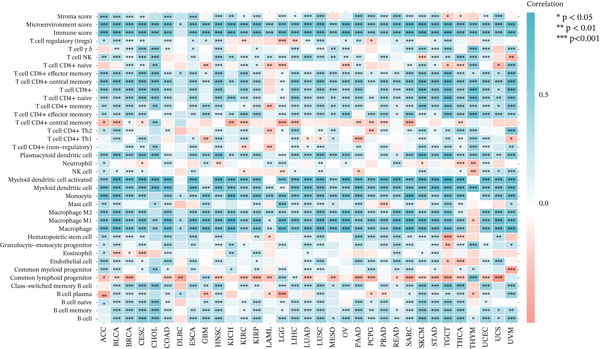
(b)

Figure 3Correlation between TIPE2 expression and immune checkpoints, as well as the immune regulators MSI and TMB. (a) TIPE2 expression showed a significant correlation with immune checkpoint genes. (b–c) Pan‐cancer analysis of the correlation between TIPE2 expression and the immunomodulators MSI (b) and TMB (c).

**Figure:**
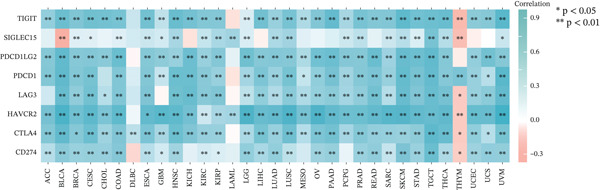
(a)

**Figure:**
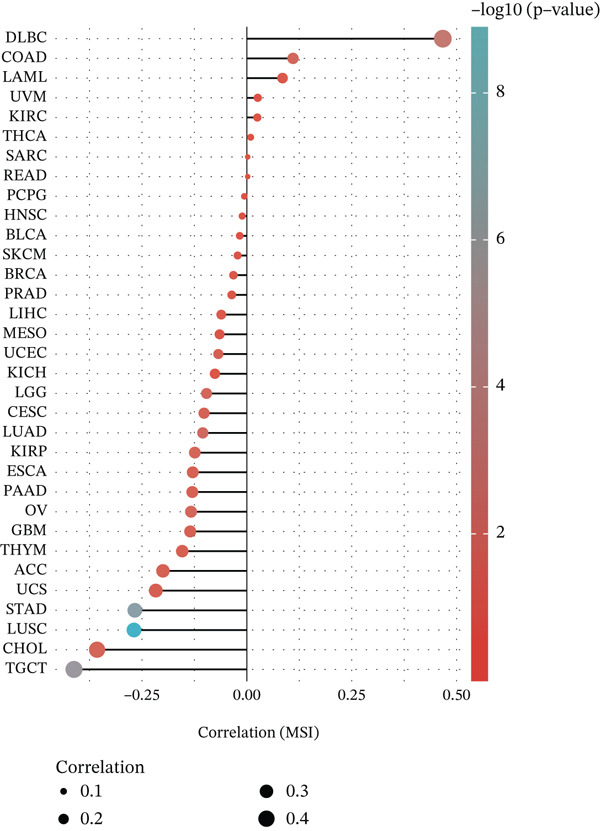
(b)

**Figure:**
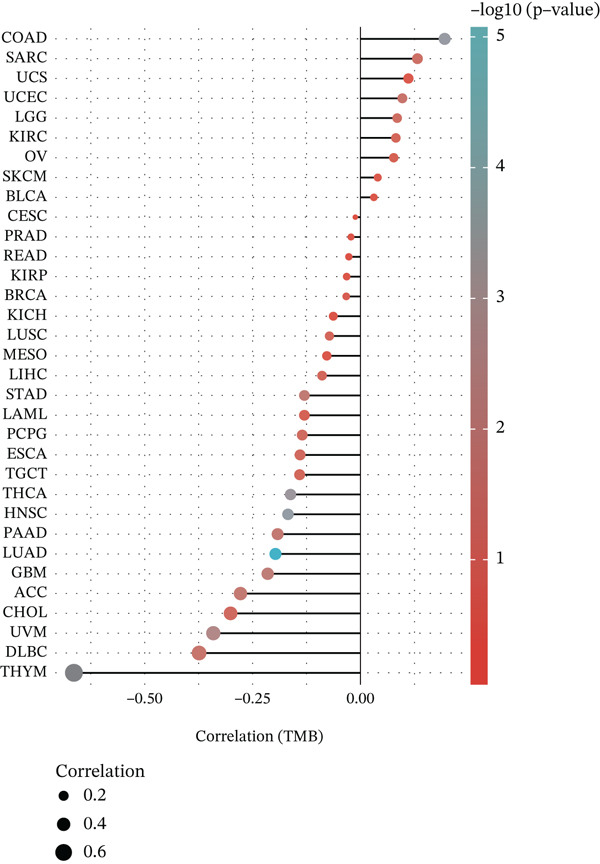
(c)

### 3.3. Mutation and Methylation of TIPE2

We analyzed TIPE2 alterations across various cancers using the cBioPortal database and identified alterations in TIPE2 in 4% (457/10,967) of pan‐cancer patients. Moreover, analysis of the alteration frequencies of TIPE2 genes across various tumor types revealed that liver hepatocellular carcinoma (11.02%), lung adenocarcinoma (9.36%), and breast invasive carcinoma (9.13%) exhibited the highest alteration rates. Notably, the most prevalent alteration of the TIPE2 gene is amplification (Figure 4a). Aberrant DNA methylation facilitates tumorigenesis and progression [35]. Therefore, we utilized the UALCAN database to investigate the methylation levels of TIPE2 across various cancers and corresponding tissues [36]. Our results demonstrated that TIPE2 methylation levels were significantly reduced in 14 tumor tissues, including BLCA, BRCA, and CHOL, compared with normal tissues (Figure 4b).

Figure 4Mutation and methylation of TIPE2. (a) A pan‐cancer analysis of the likelihood and types of TIPE2 mutations. (b) Differences in TIPE2 methylation between tumor and normal tissues.

**Figure:**
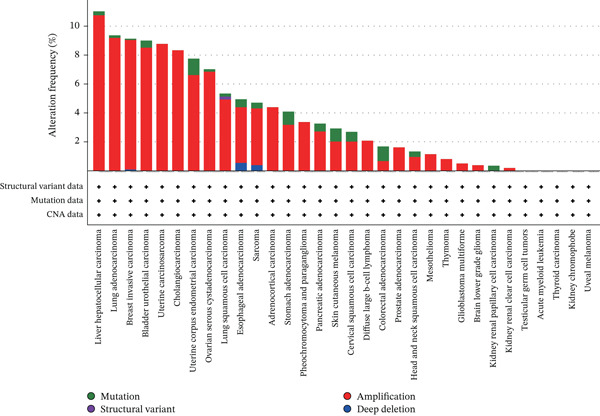
(a)

**Figure:**
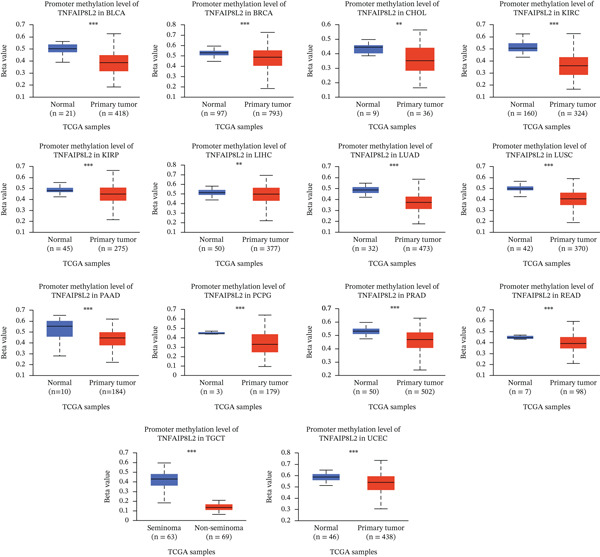
(b)

### 3.4. The Enrichment Pathway of TIPE2

To explore the mechanism of TIPE2 in tumor progression, we utilized the TCGA database to retrieve genes involved in key pathways and conducted analyses using GSEA software and Spearman correlation to compare gene expression and pathway scores [37]. The enrichment pathways were ranked according to the normalized enrichment score (NES). As shown in Figure 5a, the representative enriched pathways included hematopoietic cell lineage, the intestinal immune network for IgA production, and primary immunodeficiency [31]. We also evaluated the enrichment pathways associated with decreased TIPE2 expression, including the metabolism of xenobiotics by cytochrome P450, steroid hormone biosynthesis, and retinol metabolism (Figure 5b).

Figure 5The enrichment pathway associated with TIPE2. (a) Representative enrichment pathways of TIPE2. (b) Bubble diagram illustrating the enrichment pathways associated with TIPE2 expression.

**Figure:**
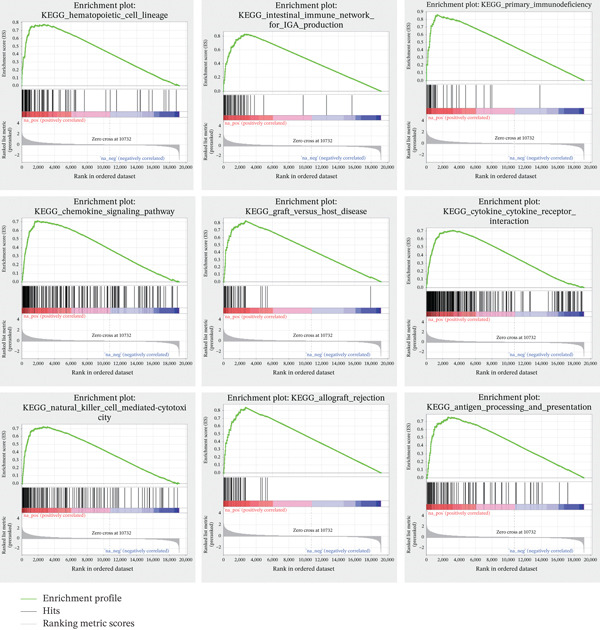
(a)

**Figure:**
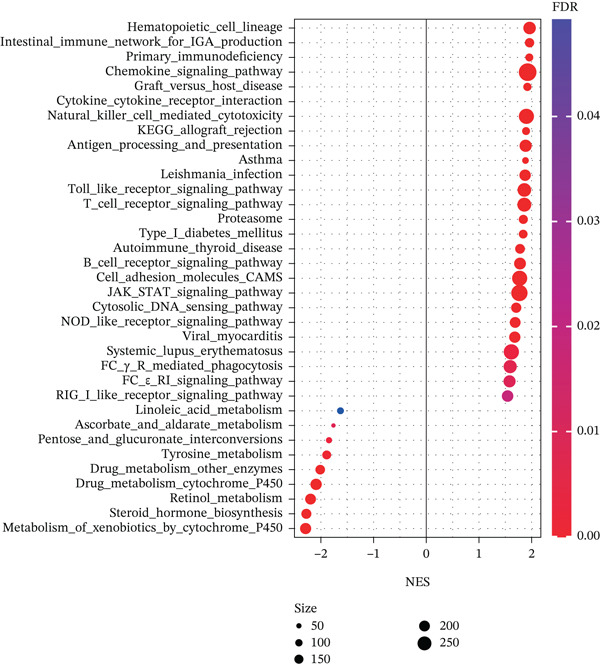
(b)

### 3.5. The Relationship Between TIPE2 Expression and Survival Outcomes in Tumor Patients

We further evaluated the association between TIPE2 expression and cancer patient prognosis using Kaplan–Meier survival analysis [38]. As shown in Figure 6a, high TIPE2 expression was associated with improved DFS in ACC and CHOL, as well as enhanced OS in BRCA, CESC, DLBC, SARC, and SKCM. Conversely, elevated TIPE2 expression correlated with poorer DFS in PRAD and reduced OS in LGG and UVM (*p* < 0.05) [16, 32]. In addition, we performed Cox regression analysis on RNA‐seq data and corresponding clinical pan‐cancer information obtained from the TCGA database using R software. As shown in Figure 6b, our Cox regression analysis suggested that TIPE2 expression was significantly associated with DFS in CESC and ESCA, as well as OS in BRCA, LAML, LGG, SARC, SKCM, THYM, and UVM (*p* < 0.05).

Figure 6The relationship between TIPE2 expression and tumor patient survival. (a) Kaplan–Meier survival analysis demonstrated that differential TIPE2 expression in tumors predicted the prognoses of cancer patients. (b) Cox regression analysis showing the correlation between TIPE2 expression and the prognosis of tumor patients.

**Figure:**
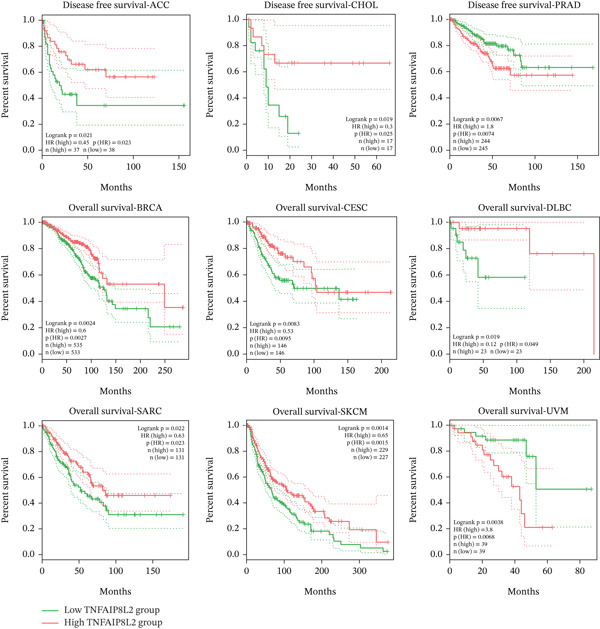
(a)

**Figure:**
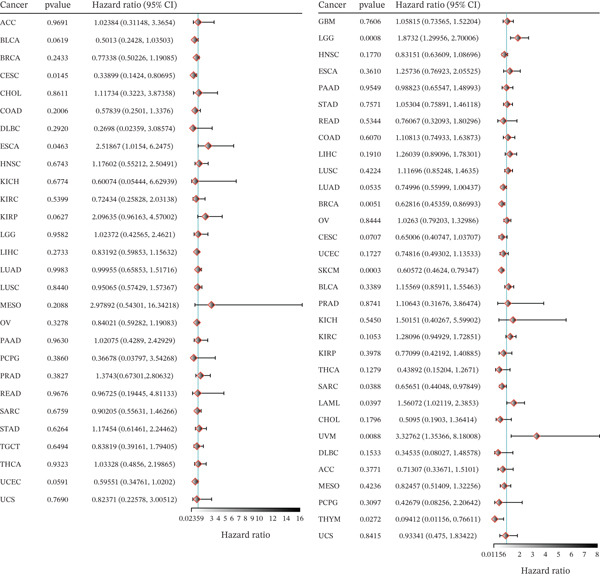
(b)

### 3.6. Correlation Between TIPE2 Expression and Tumor Pathological Stage

**Figure 7:**
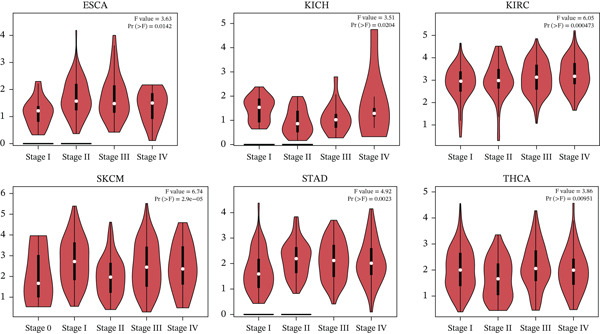
A comprehensive pan‐cancer analysis of the correlation between TIPE2 expression and tumor pathological stage.

The pathological stage of the tumor indicates the degree of tumor malignancy and serves as a critical prognostic factor for the patient. Therefore, we utilized GEPIA2 to analyze the correlation between TIPE2 expression and tumor pathological stage across 24 cancer types, identifying statistically significant associations (*p* < 0.05) in ESCA, KICH, KIRC, SKCM, STAD, and THCA (Figure 7) [21, 39, 40].

## 4. Discussion

In this study, we conducted a pan‐cancer analysis to elucidate the multifaceted role of TIPE2 within the TME. TIPE2 is a member of the TIPE family and exhibits high sequence homology with other members of this family [11, 12]. To explore the role of TIPE2, we first compared TIPE2 expression across various human organs and found significantly increased levels in both central and peripheral immune organs compared with other tissues. These findings are corroborated by previous studies indicating that TIPE2 plays a role in the body′s immune response. Subsequently, we analyzed TIPE2 expression in 33 tumor tissues. Our results showed increased TIPE2 expression in various tumor tissues. Based on previous data analysis, we concluded that increased TIPE2 expression levels in tumors are likely associated with the body′s immune response [7]. We further used HPA to analyze TIPE2 expression across various cell types and found high TIPE2 expression levels in T cells, B cells, natural killer cells, monocytes, and other immune cells. This further indicated a significant association between increased TIPE2 expression in tumor tissues and the infiltration of tumor immune cells. This aligns with relevant studies, further indicating that TIPE2 is closely related to immune cells [31]. However, TIPE2 expression varies among immune cells, and the tumor immune infiltrating cells (TIICs) identified in different tumor tissues differ, necessitating further investigation to elucidate the specific mechanism by which TIPE2 affects the immune response. To this end, our analysis of the relationship between TIPE2 expression and tumor immune cell infiltration suggests that TIPE2 expression is frequently positively correlated with the infiltration of immune cells [31]. It is worth noting that, among the 33 tumor types studied, TIPE2 was not highly associated with immune cell infiltration in DLBC and LAML, which may be partly attributed to the inherently high TIPE2 expression levels in immune and blood cells. Pan‐cancer analyses of immune checkpoints reinforce these findings, indicating that TIPE2 may play a role in the immune regulatory network involved in tumor immune evasion. TIPE2 serves as a negative regulatory factor for both innate and adaptive immunity, effectively inhibiting the excessive activation of TCRs and TLR signaling pathways. TIPE2 has been reported to negatively regulate the PI3K/Akt and NF‐*κ*B signaling pathways, both of which are known to contribute to the regulation of PD‐L1 expression within the TME [41, 42].

Specifically, the phosphatidylinositol‐binding property of TIPE2 enables it to compete for PtdIns(4,5)P_2_ and PtdIns(3,4,5)P_3_, thereby attenuating the recruitment of downstream kinases such as Akt and the subsequent phosphorylation of IKK complexes. By suppressing NF‐*κ*B activation, TIPE2 may indirectly modulate the transcriptional regulation of genes associated with immune checkpoints. Therefore, the strong correlation between TIPE2 and checkpoint markers observed in our analysis may represent a compensatory or homeostatic feedback response to heightened immune activation. Tumor cells have exploited this process to promote immune evasion [42, 43]. Furthermore, our GSEA results also demonstrate extensive enrichment of TIPE2 in immune‐related pathways. Studies suggest that TIPE2 can affect the migration of white blood cells and that an increase in TIPE2 levels is directly correlated with elevated inflammatory‐related factors. These findings suggest that TIPE2 plays a unique role within the TME, especially in immune‐related cells [4, 10, 32, 33, 44–46].

In addition to its role in molecular signal transduction, TIPE2 directly regulates tumor progression by modulating the functional states of specific immune cell subsets. Recent evidence indicates that TIPE2 is the principal regulatory factor for the polarization of myeloid‐derived suppressor cells (MDSCs). Tumor‐derived IL‐6 and reactive oxygen species (ROS) stimulate the expression of TIPE2 in MDSCs through the STAT3 signaling pathway. The elevation of TIPE2 levels subsequently promotes the immunosuppressive phenotype of MDSCs, thereby impairing the antitumor activity of cytotoxic T lymphocytes (CTLs) and facilitating tumor progression [32, 47].

Subsequently, we examined the alterations in TIPE2. The transformation of normal cells into malignant ones is typically accompanied by gene mutations, making the identification of mutated oncogenes essential for the development of effective cancer detection and treatment strategies. The alteration analysis of TIPE2 revealed that (1) alterations in the TIPE2 gene were present in 4% of tumor patients, and (2) amplification was the most prevalent mutation type. The development and utilization of drugs for these patients contribute significantly to the advancement of precision medicine. DNA methylation plays a crucial role in regulating gene expression, and abnormal DNA methylation is frequently associated with cancer. Therefore, DNA methylation detection represents a novel tool that has the potential to enhance the accuracy of pathological diagnoses [36]. Our results show that TIPE2 was aberrantly methylated in 14 tumors, including BLCA, BRCA, and CHOL. However, a search of the TISIDB database (http://cis.hku.hk/TISIDB/index.php) did not yield any information regarding TIPE2‐associated oncology therapeutics. The significant correlations observed between TIPE2 expression, tumor immune cell infiltration levels, and immune checkpoint gene expression in this study underscore the need for further research on TIPE2 expression and immune cell infiltration across diverse cancer populations, with the aim of advancing TIPE2‐targeted therapeutic strategies to enhance the effectiveness of existing immunotherapies. TIPE2 possesses dual potential as both a predictive biomarker and a therapeutic target. In terms of biomarkers, the combination of TIPE2 with tumor mutation burden (TMB) or microsatellite instability (MSI) status facilitates more precise patient screening for immune checkpoint inhibitors by identifying cases susceptible to MDSC‐mediated resistance. In terms of treatment, the unique hydrophobic pocket of TIPE2 offers the possibility for the development of small molecule inhibitors. Additionally, silencing TIPE2 in adoptive cell therapy, such as CAR‐NK cells, represents a highly promising strategy that can simultaneously reactivate lymphoid effector cells and reverse myeloid‐mediated immune suppression [32, 48, 49].

Finally, we employed prognostic prediction methods to examine the association between TIPE2 and tumor pathological staging, as well as survival outcomes in tumor patients. In ACC, CHOL, BRCA, CESC, DLBC, SARC, and SKCM, our data indicate that high TIPE2 expression is associated with improved survival, whereas in PRAD and UVM, elevated TIPE2 expression corresponds to poorer survival. Related studies indicate that TIPE2 may suppress breast cancer by inhibiting the phosphorylation of AKT and p38. Furthermore, consistent with our findings, multiple studies have demonstrated that TIPE2 expression is upregulated and may serve as a prognostic indicator in DLBC. Similarly, other studies indicate that TIPE2 exerts an inhibitory effect in lung cancer, oral tongue squamous cell carcinoma, gastric cancer, esophageal cancer, and various other tumors, thereby indicating an additional role for TIPE2 in predicting the prognosis of tumor patients [8, 17, 46, 50].

In summary, this study demonstrates that TIPE2 is a protein broadly expressed in immune cells [19]. Based on its expression pattern, TIPE2 has been implicated in diverse immune‐inflammatory processes and multiple immune‐related diseases, underscoring the potential clinical relevance of its measurement [51]. Importantly, our pan‐cancer analyses identify TIPE2 as an immune‐enriched regulator whose expression closely correlates with immune cell infiltration and checkpoint gene alterations across the majority of tumor types, highlighting its mechanistic significance in modulating the tumor immune microenvironment. Future research will predominantly utilize TIPE2 knockout mouse models and in vitro coculture experiments to validate these observations across all cancer types. In addition, the application of advanced technologies, such as spatial and single‐cell profiling, is also warranted.

## 5. Conclusions

Our integrated pan‐cancer multiomics analysis across 33 tumor types indicates that TIPE2 is an immune‐enriched regulator, primarily expressed in blood and immune cells, and is frequently dysregulated in tumors. TIPE2 expression exhibits broad, tumor‐specific associations with immune infiltration and immune checkpoint gene expression, and is associated with key immunologic pathways as demonstrated by GSEA, supporting its role in modulating the TME. Genomic and epigenetic alterations of TIPE2 are relatively common (occurring in approximately 4% of cases, predominantly amplifications), and TIPE2 hypomethylation has been observed in multiple malignancies, suggesting additional regulatory mechanisms. Importantly, TIPE2 possesses prognostic significance with varying effects across different cancers, being associated with favorable outcomes in several cohorts such as BRCA, SKCM, CESC, ACC, and CHOL for DFS, but with unfavorable outcomes in others, including LGG, UVM, and PRAD for DFS. Collectively, these findings identify TIPE2 as a promising biomarker for risk stratification and immunotherapy‐driven patient selection, as well as a potential therapeutic target for modulating immune suppression. Future studies should prioritize mechanistic validation through coculture systems, spatial and single‐cell profiling, TIPE2 genetic perturbation models, and prospective clinical studies to define its predictive value for checkpoint blockade response.

## Author Contributions

Hui Cao, Wenyu Jia, and Hao Yang contributed equally to this work and are considered co‐first authors. Zequn Li and Yanbing Zhou designed the study. Zequn Li acquired funding. Hui Cao, Wenyu Jia, and Hao Yang wrote the original draft. Yuling Zhang and Shougen Cao contributed to the figures. Zequn Li, Yanbing Zhou, and Shougen Cao reviewed the manuscript.

## Funding

This study was supported by Young Talent of Lifting Engineering for Science and Technology in Shandong, China (SDAST2024QTA032) and Wu Jieping Medical Foundation Clinical Research Special Fund (320.6750.2024‐17‐41).

## Disclosure

All authors read and approved the final manuscript.

## Ethics Statement

The authors have nothing to report.

## Conflicts of Interest

The authors declare no conflicts of interest.

## Data Availability

The data that support the findings of this study are openly available in The Cancer Genome Atlas (TCGA) (https://portal.gdc.cancer.gov/), the Genotype‐Tissue Expression (GTEx) project (https://gtexportal.org/), the cBioPortal (https://www.cbioportal.org/), the Human Protein Atlas (HPA) (https://www.proteinatlas.org/), and the UALCAN portal (http://ualcan.path.uab.edu/).
